# Supplementary material for: Friendship habits questionnaire: A measure of group- versus dyadic-oriented socializing styles
Source: PLoS One. 2023 Jun 28;18(6):e0285767. doi: 10.1371/journal.pone.0285767 (PMC10306221; doi:10.1371/journal.pone.0285767)
Supplement: S2 Fig — (DOCX) [file pone.0285767.s002.docx]

Figure S2

*Study 1: Scree Plot Showing Eigenvalues of Each Principal Component for the varimax rotation*


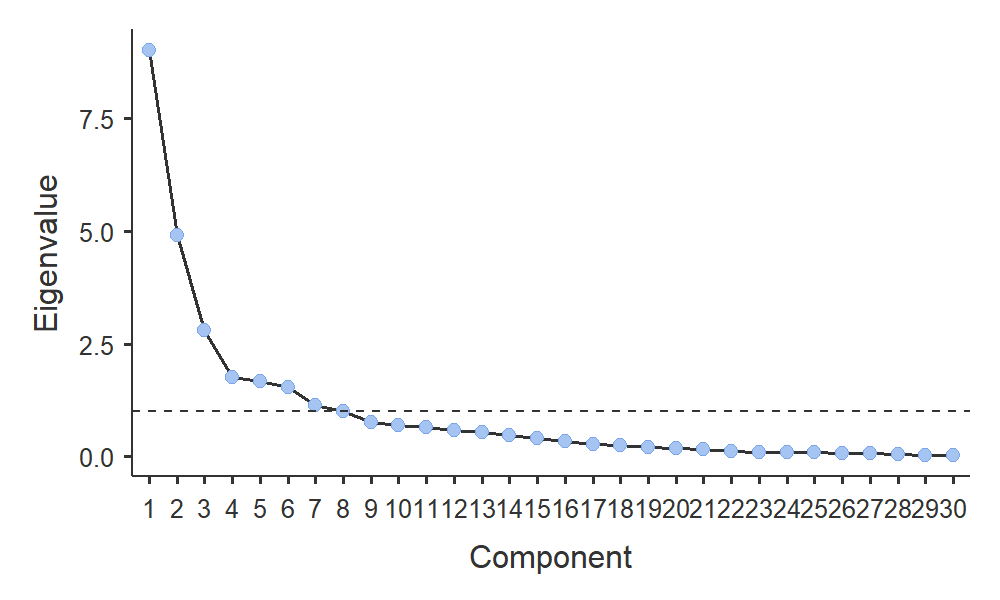


Created by the jamovi project (2023) on R. jamovi. (Version 1.2) [Computer Software].
