# Supplementary material for: Friendship habits questionnaire: A measure of group- versus dyadic-oriented socializing styles
Source: PLoS One. 2023 Jun 28;18(6):e0285767. doi: 10.1371/journal.pone.0285767 (PMC10306221; doi:10.1371/journal.pone.0285767)
Supplement: S1 Table — (DOCX) [file pone.0285767.s003.docx]

Table S1

*Study 1: Descriptive Statistics and Component Loadings for the PCA Analysis with varimax rotation.*

|  |  |  |  | Component Loading | | | | | |
| --- | --- | --- | --- | --- | --- | --- | --- | --- | --- |
|  |  |  |  | 1 | 2 | 3 | 4 | 5 | 6 |
| **Dimension** | **FHQ Items** | **M** | **SD** | EXTR | INTIM | COMP | POS GROUP ID | NEG GROUP ID | CONT |
| Extraversion  (*M* = 3.02, *SD* = .41) | E1 I am outgoing and sociable when I am in a larger group of friends | 3.10 | 1.01 | **.86** |  |  |  |  |  |
|  | E2 I am talkative when I am in larger group of friends | 3.04 | 1.14 | **.81** |  |  |  |  |  |
|  | E3 I am reserved when I am in larger group of friends^Δ^ | 2.96 | 1.17 | **-.63** |  |  |  | .55 |  |
|  | E4 I am full of energy when I am in larger group of friends | 2.96 | 1.08 | **.69** |  |  |  |  |  |
|  | E5 I tend to be quiet when I am in larger group of friends^Δ^ | 2.98 | 1.16 | **-.66** |  |  |  | .40 |  |
|  | E6 I have an assertive personality | 3.04 | 1.00 | **.69** |  |  |  |  | -.32 |
|  | E7 I am sometimes shy and inhibited in larger group of friends^Δ^ | 3.08 | 1.15 | **-.51** |  |  |  | .33 |  |
|  | E8 I generate a lot of enthusiasm when I am in larger group of friends | 3.00 | 1.10 | **.78** |  |  |  |  |  |
| Intimacy | I1 My friends and I always tell each other our problems^Δ^ | 3.51 | 1.06 |  | **.81** |  | .30 |  |  |
| (*M* = 3.53^***^, *SD* = .78) | I2 My friends and I talk about the things that make us sad^Δ^**Error! Bookmark not defined.** | 3.49 | 1.06 |  | **.79** |  |  |  |  |
|  | I3 I tell my friends when I am mad about something that happened to me^Δ^ | 3.67 | 0.90 |  | **.57** |  |  |  | -.33 |
|  | I4 My friends and I tell each other secrets^Δ^ | 3.67 | 1.01 |  | **.75** |  |  |  |  |
|  | I5 My friends and I tell each other private things^Δ^ | 3.98 | 0.83 |  | **.71** |  |  |  |  |
|  | I6 My friends and I talk about how to make ourselves feel better if we are mad at each other^Δ^ | 2.86 | 1.17 | .34 | **.67** |  |  |  |  |
| Competitiveness | C1 I like competition among friends | 2.31 | 1.28 |  |  | **.81** |  |  |  |
| (Enjoyment of | C2 I enjoy competing against a friend | 2.55 | 1.19 |  |  | **.88** |  |  |  |
| Competition) | C3 I don't like competing against a friend^Δ^ | 3.49 | 1.29 |  |  | **-.76** |  | .43 |  |
| (*M* = 2.81^†^, *SD* = .66) | C4 I am a competitive individual | 2.90 | 1.25 |  |  | **.65** |  |  |  |
| Competitiveness  (Contentiousness) | C5 I will do almost anything to avoid an argument with friends^Δ^ | 3.14 | 1.08 |  |  |  |  |  | **.90** |
| (*M* = 3.19, *SD* = .82) | C6 I try to avoid arguments with friends^Δ^ | 3.37 | 1.04 |  |  | -.32 |  |  | **.80** |
|  | C7 I often remain quiet rather than risk hurting another friend's feelings^Δ^ | 3.06 | 0.88 | -.51 |  |  |  |  | **.53** |
| Group Identification | GP1 I am glad when I belong to a friendship group | 3.61 | 1.02 |  |  |  | **.42** |  |  |
| (Positive) | GP2 I identify with a friendship group | 3.45 | 1.14 |  |  |  | **.79** | -.36 |  |
| (*M* = 3.47^***^, *SD* = .84) | GP3 I feel strong ties to a friendship group | 3.39 | 1.15 | .34 | .41 |  | **.70** |  |  |
|  | GP4 I think friendship groups work well together | 3.49 | 0.85 |  |  |  | **.75** |  |  |
|  | GP5 I see myself as an important part of a friendship group | 3.39 | 1.10 |  | .33 |  | **.69** |  |  |
| Group Identification | GN1 I feel held back in friendship groups^Δ^ | 2.39 | 1.08 |  |  |  | -.32 | **.59** |  |
| (Negative) | GN2 I do not consider a friendship group to be important^Δ^ | 2.31 | 1.16 |  | -.30 | .31 |  | **.33** |  |
| (*M* = 2.40^***^, *SD* = .92) | GN3 I do not fit in well with other members of friendship groups^Δ^ | 2.51 | 1.18 |  |  |  |  | **.84** |  |
|  | GN4 I feel uneasy with members of friendship groups^Δ^ | 2.41 | 1.14 |  |  |  |  | **.62** |  |

*Note. EXTR = Extraversion,* *INTIM = Intimacy, COMP = Competitiveness, POS GROUP ID = Positive Group Identification, NEG GROUP ID = Negative Group Identification, CONT = Contentiousness. Component loadings smaller than .30 are omitted and highest component loadings for each item are in boldface. The percentage of variance for each component was as follows: EXTR = 17.09%, INTIM = 13.43%, COMP = 10.66%, POS GROUP ID = 10.05%, NEG GROUP ID = 9.41%, and CONT = 7.49%. In total, 68.12% of variance was explained. Δs denote reverse-scored FHQ items in Column 2, but please note that intimacy scores were unreversed in the final version of the FHQ (see Study 2 and 3 for clarifications).*  *Asterisks in Column 1 denote values significantly different from 3, the scale midpoint,* ^†^ *p < .10, ^*^p < .05, ^**^p < .01, and ^***^p < .001. Differences were tested using one-sample t-tests, except for Extraversion. The distribution of this component departed from normality, W(48) = .93, p < .008 and we used a One-Sample Wilcoxon Signed Rank Test..*
