# Supplementary material for: Friendship habits questionnaire: A measure of group- versus dyadic-oriented socializing styles
Source: PLoS One. 2023 Jun 28;18(6):e0285767. doi: 10.1371/journal.pone.0285767 (PMC10306221; doi:10.1371/journal.pone.0285767)
Supplement: S2 Table — (DOCX) [file pone.0285767.s004.docx]

Table S2

*Study 2: Factor Loadings the Four-Factor Theoretical Model (Extraversion, Competitiveness, Intimacy and Group Identification)*

| Factor | Item | Estimate | Completely Standardized Solution | SE | p |
| --- | --- | --- | --- | --- | --- |
| Extraversion | E1 | 1.22 | .85 | .07 | <.001 |
|  | E2 | 1.24 | .87 | .07 | <.001 |
|  | E3 | 1.17 | .83 | .07 | <.001 |
|  | E4 | 1.09 | .79 | .07 | <.001 |
|  | E5 | 1.23 | .82 | .07 | <.001 |
|  | E6 | .57 | .43 | .08 | <.001 |
|  | E7 | .98 | .67 | .08 | <.001 |
|  | E8 | .94 | .74 | .06 | <.001 |
| Competitiveness | C1 | 1.52 | .86 | .06 | <.001 |
|  | C2 | 1.71 | .95 | .06 | <.001 |
|  | C3 | 1.37 | .78 | .08 | <.001 |
|  | C4 | .96 | .55 | .09 | <.001 |
|  | C5 | .46 | .27 | .10 | <.001 |
|  | C6 | .45 | .28 | .10 | <.001 |
|  | C7 | .39 | .24 | .10 | <.001 |
| Intimacy | I1 | .97 | .75 | .08 | <.001 |
|  | I2 | .89 | .64 | .08 | <.001 |
|  | I3 | .69 | .58 | .08 | <.001 |
|  | I4 | 1.08 | .84 | .07 | <.001 |
|  | I5 | .99 | .80 | .08 | <.001 |
|  | I6 | .65 | .44 | .08 | <.001 |
| Group Identification | GP1 | .01 | .62 | .00 | <.001 |
|  | GP2 | .01 | .77 | .00 | <.001 |
|  | GP3 | .01 | .78 | .00 | <.001 |
|  | GP4 | .01 | .61 | .00 | <.001 |
|  | GP5 | .01 | .71 | .00 | <.001 |
|  | GN1 | .01 | .51 | .00 | <.001 |
|  | GN2 | .01 | .54 | .00 | <.001 |
|  | GN3 | .01 | .58 | .00 | <.001 |
|  | GN4 | .01 | .45 | .00 | <.001 |
| Friendship Styles | Extraversion | .68 | .56 | .09 | <.001 |
|  | Competitiveness | .17 | .17 | .07 | .012 |
|  | Intimacy | -.56 | -.49 | .08 | <.001 |
|  | Group Identification | 111.93 | 1.00 | 12.29 | <.001 |
