# Supplementary material for: Friendship habits questionnaire: A measure of group- versus dyadic-oriented socializing styles
Source: PLoS One. 2023 Jun 28;18(6):e0285767. doi: 10.1371/journal.pone.0285767 (PMC10306221; doi:10.1371/journal.pone.0285767)
Supplement: S3 Table — (DOCX) [file pone.0285767.s005.docx]

**Table S3**

*Study 2: Factor Correlations for the Four-Factor Theoretical Model.*

|  | 1 | 2 | 3 | 4 |
| --- | --- | --- | --- | --- |
| 1. Extraversion | - |  |  |  |
| 2. Competitiveness | .18^**^ | - |  |  |
| 3. Intimacy^Δ^ | -.27^**^ | -.01 | - |  |
| 4. Group Identification | .56^***^ | .17^**^ | -.49^***^ | - |

*Note. Δ for reversed scored factor.*
