# Supplementary material for: Friendship habits questionnaire: A measure of group- versus dyadic-oriented socializing styles
Source: PLoS One. 2023 Jun 28;18(6):e0285767. doi: 10.1371/journal.pone.0285767 (PMC10306221; doi:10.1371/journal.pone.0285767)
Supplement: S4 Table — (DOCX) [file pone.0285767.s006.docx]

Table S4

*Study 2: Factor Loadings the Six-Factor Model (Extraversion, Enjoyment of Competition, Contentiousness, Intimacy, Positive Group Identification and Negative Group Identification)*

| Factor | Item | Estimate | Completely Standardized Solution | SE | p |
| --- | --- | --- | --- | --- | --- |
| Extraversion | E1 | 1.13 | .85 | .08 | <.001 |
|  | E2 | 1.15 | .87 | .08 | <.001 |
|  | E3 | 1.09 | .83 | .08 | <.001 |
|  | E4 | 1.01 | .79 | .08 | <.001 |
|  | E5 | 1.15 | .82 | .08 | <.001 |
|  | E6 | .53 | .43 | .08 | <.001 |
|  | E7 | .91 | .67 | .08 | <.001 |
|  | E8 | .87 | .73 | .08 | <.001 |
| Enjoyment of Competition | C1 | 1.50 | .85 | .07 | <.001 |
|  | C2 | 1.74 | .97 | .06 | <.001 |
|  | C3 | 1.36 | .77 | .08 | <.001 |
|  | C4 | .96 | .55 | .09 | <.001 |
| Contentiousness | C5 | 1.30 | .76 | .09 | <.001 |
|  | C6 | 1.32 | .81 | .10 | <.001 |
|  | C7 | .95 | .58 | .10 | <.001 |
| Intimacy | I1 | .96 | .75 | .08 | <.001 |
|  | I2 | .88 | .64 | .08 | <.001 |
|  | I3 | .69 | .58 | .08 | <.001 |
|  | I4 | 1.07 | .83 | .07 | <.001 |
|  | I5 | .99 | .80 | .08 | <.001 |
|  | I6 | .64 | .44 | .08 | <.001 |
| Positive Group Identification | GP1 | .41 | .62 | .08 | <.001 |
|  | GP2 | .67 | .78 | .12 | <.001 |
|  | GP3 | .67 | .81 | .12 | <.001 |
|  | GP4 | .40 | .62 | .07 | <.001 |
|  | GP5 | .60 | .72 | .10 | <.001 |
| Negative Group Identification | GN1 | .45 | .61 | .11 | <.001 |
|  | GN2 | .44 | .52 | .10 | <.001 |
|  | GN3 | .51 | .71 | .12 | <.001 |
|  | GN4 | .42 | .60 | .11 | <.001 |
| Friendship Styles | Extraversion | .84 | .64 | .13 | <.001 |
|  | Competitiveness | .16 | .16 | .08 | .031 |
|  | Contentiousness | .14 | .14 | .10 | .163 |
|  | Intimacy | -.58 | -.50 | .09 | <.001 |
|  | Positive Group ID | 1.58 | .85 | .37 | <.001 |
|  | Negative Group ID | 1.82 | .88 | .51 | <.001 |
