# Supplementary material for: Friendship habits questionnaire: A measure of group- versus dyadic-oriented socializing styles
Source: PLoS One. 2023 Jun 28;18(6):e0285767. doi: 10.1371/journal.pone.0285767 (PMC10306221; doi:10.1371/journal.pone.0285767)
Supplement: Attachment — Submitted filename: Review report - PONE-D-22-25565.docx [file pone.0285767.s007.docx]

Reviewer reports

Thanks for inviting me to review the manuscript “Friendship Habits Questionnaire: A measure of group- versus dyadic-oriented socializing preferences”. The purpose of the manuscript is to present the process of development of a new questionnaire to assess friendship habits with the focus on preferences of amount of persons friendship is experiences with. The authors call it “group versus dyadic- oriented”. The manuscript contains three sequential studies which describes the refinement of the underlying constructs and first psychometric properties to validate the new measure FHQ (Friendship Habit Questionnaire) such as construct validity or reliability.

The overall flow of the paper is solid and organized in a fluent and scientific manner and thus informative. The paper is in general quite long, as one could expect in describing three validation studies. As friendship is such an important part of life, a variation of assessments with distinct underlying concepts are needed to understand this complex phenome better. Therefor the authors can be complemented for their efforts and hard work to present the development of the FHQ.

Despite the accolades, there are several suggestions to improve the strength of the manuscript.

**Abstract:**

It should be explicit that this is a new measure and that the three performed studies investigated psychometric properties and refinement of the underlying construct.

It is further confusing to first read about competitiveness and later in the results about other dimensions. It might be more important to present the overall aim of all three studies.

**Introduction:**

Please explain more in detail why this new measure is needed (line 61- 65 is too short)

Line 68: it is definitely needed to explain how you came to the construct with the four dimensions you started with and made factor analysis.

Please insert a clear overall research question (Line 109) instead of describing the overall examination.

Line 110. Which previous findings? Please insert again references.

Line 116-124:	Instead of describing the organization of the three studies, I would prefer a figure (type flowchart? ) to support readers orientation.

**Study 1**

Please organize each study (1-3) along research questions, hypothesis followed (as you did) by methods (participants, recruitment, material) and analysis. This would make it much easier to retrain from repetition such as in line 128 – 131.

I propose to put the description of the FHQ with the original underlying concepts derived from other measures in the introduction (line 150 – 168). Then each of the sequential studies are shorter and easier to understand.

Line 170 – 174:	differentiate between analysis and results instead of mixing them. This is for me as reader confusing.

Table 1 is informative, but the Asterisks confusing with p>0.5. So I suggest using another graphical solution to indicate reverse-scores FHQ items.

Discussion Study 1: very informative

**Study 2**

See suggestion of organization of study 1.

Line 242 -245	redundancy, instead make the aims and /or the different research questions of study 2 explicit (may be also possible to put into the flowchart/figure?)

Line 267: Again, separate analysis and results.

Table 4: I miss explanation of the Friendship preferences. Are these summary scores?

Table 6: the first line does not fit with the following ones. Please adjust.

**Study 3**

Line 412 and 416: give a methodological reason for the attention check.

Line 412:	please explain why you changed the options to describe the preference of friendship habits as compared to study 2.

Line 425: I suggest deleting the emotion recognition task if not explained.

**General Discussion**

I disagree with the main aim to validate the FHQ. To my understanding, the three sequential studies develop the FHQ by examining and adapting the construct to achieve best validity and a small part of reliability. As validation of a measurement are somehow much more than you did (e.g. concurrent validity, age and gender validity….) , I suggest to stay here modest.

Line 540: I expect here more suggestions how to use the FHQ and practical implications. Asking directly are 4 main questions, while the FHQ has 30! Line 544 What prosper utility see the authors?

Line 550: I think you should reflect on more limitations of your work. One is the participants of these studies: first their age rang is quite small and secondly the gender inequality may have an influence on your results. Please also reflect on the influence the Covid lockdown might have on the results. Another limitation is that reliability is not measured with test-retest reliability or stability over time.

Again, the authors should be commended on their time and effort develop a new measurement I the field of friendship evaluation. Continued revisions will strengthen the summaries of this early stage manuscript document.
