# Supplementary material for: Friendship habits questionnaire: A measure of group- versus dyadic-oriented socializing styles
Source: PLoS One. 2023 Jun 28;18(6):e0285767. doi: 10.1371/journal.pone.0285767 (PMC10306221; doi:10.1371/journal.pone.0285767)
Supplement: Attachment — Submitted filename: Response to Reviewers.docx [file pone.0285767.s008.docx]

January 24, 2023

Dear Professor Fang Wang,

**Re: Submission of the revised manuscript “Friendship Habits Questionnaire: A measure of group- versus dyadic-oriented socializing styles”**

Please find enclosed the revision of our manuscript “Friendship Habits Questionnaire: A measure of group- versus dyadic-oriented socializing preferences” (new title above). The authors are: Philip Howlett (phowlett01@qub.ac.uk), Tomas Jungert (tomas.jungert@psy.lu.se), Gulseli Baysu (G.Baysu@qub.ac.uk), Anthony Atkinson (a.p.atkinson@durham.ac.uk), and Magdalena Rychlowksa, (M.Rychlowska@qub.ac.uk),

We thank the reviewers for their positive feedback and appreciate their constructive comments that greatly helped in improving the article. Below we address each of the points that were raised.

Please do not hesitate to contact us if there are any outstanding issues. Thank you for your time and consideration.

Sincerely,

Philip Howlett

School of Psychology

Queen’s University Belfast

**Reviewer 1**

- *The authors report three studies that examined individual differences associated with socializing in pairs versus groups and put forward the Friendship Habits Questionnaire (FHQ) for measuring socializing preferences (either dyad oriented or group oriented). (…) After addressing minor concerns, I would consider it publication-worthy.*

**Thank you so much for your positive comments and thorough, excellent summary of the research.**

- *Page 4. Lines 65–68: “Here we present the Friendship Habits Questionnaire (FHQ), a new tool measuring whether one’s socializing preferences are more dyadic versus group-oriented based on individual differences in needs for intimacy, competitiveness, group identification, and extraversion.”*

*Comment: The order of individual difference variables is listed as needs for intimacy, competitiveness, group identification and extraversion and subsequently described in more detail in the following paragraphs in the order extraversion, group identification, intimacy, competitiveness.*

*The authors may consider bringing these into harmony by changing the order in lines 65–68 to correspond with the order of subsequent elaboration.*

**We have now changed this in line with your suggestion (see lines 85-86, page 4, in the manuscript with tracked changes).**

- *(2) Page 5. Lines 88–89. The sentence beginning with the words “Another relevant construct is intimacy, (…)” may start a new paragraph just like the other individual difference variables, instead of being combined into a single paragraph with group identification.*

**We have now started a new paragraph for intimacy (line 108, page 5).**

- *(3) Page 8. Lines 172–175. “We used varimax rotation following the procedure used by Hinkin and Tracey (41), given that we expected correlated factors, we also ran a PCA using an oblimin rotation, which showed an identical pattern of six interpretable components.”*

*Comment: Varimax rotation is an orthogonal type of rotation. In orthogonal rotation, the factors are constrained to be uncorrelated, that is, one believes the underlying factors are independent. The use of orthogonal rotation may produce misleading solutions where the factors are expected to be intercorrelated, for example in the present study, a questionnaire whose latent structure entails several interrelated dimensions of a broader construct. Fortunately, the authors have also run a PCA with oblimin rotation which yielded similar results. However, the above reference to and use of varimax should be addressed to avoid confusion.*

**The rationale for the use of varimax rotation was that this method was recommended by Hinkin and Tracey (1999) whose guidelines for questionnaire design we followed. However, we agree with your comments about the limitations of this rotation and acknowledge that the results section may be confusing for the reader. Given that a supplemental PCA using an oblimin rotation yielded an almost identical pattern of findings, we now use it as our primary analysis and have updated the Results section and Table 1 accordingly. We briefly describe the additional varimax PCA in lines 253-254, page 12, and 279 – 290, pages 13-14, when describing the decision to retain one of the items. All statistics for the varimax rotation have been moved to the Supplementary Materials and all mentions of Supplementary Materials have been updated to reflect the changes.**

- *(4) Page 10. Table 1. The second intimacy item has a notification “Error! Bookmark not defined.” that should be removed if present in the submitted manuscript.*

**This error does not appear in our version of the document and might be due to submission procedures. However, we have used Ctrl+Shift+F9 over this line (Table 1, page 15) in the table to convert it to normal text and hope that this has fixed the error. Please do highlight if this continues and we will follow up with the PLOS ONE team.**

- *(5) Page 16. Lines 310–311. “As shown in Table 4, this new model showed an acceptable fit on all indices except the relative chi-square, and was retained for further analyses.”*

*Comment: The relative chi-square is 1.88 in Table 4, and hence fits the criterion for acceptable model fit stated on page 15 line 285: “χ²/df < 2.0”.*

**Thank you for pointing out this inconsistency. We have now removed this part of the sentence (lines 430-431, page 22).**

- *(6) Page 21–22. Lines 386—389. “Although a previous study (32) showed that self-disclosure occurs more in dyads than in larger groups, this research focused on interactions between strangers, and it is unclear how self-disclosure would play out among friends.”*

*Comment: There may be some room for misunderstanding if “this research” is read as the present research or the present paper instead of the research from reference (32), which did focus on strangers instead of friendship interactions.*

**We have now reworded this sentence (see line 509, page 27).**

- *(7) Page 23. Lines 428–429. “We assessed the goodness of fit of the four-factor second-order model using the same criteria as in Study 2.”*

*Comment: In Table 7, on page 23, the SRMR and RMSEA values are missing.*

**Thank you for noticing this inconsistency. We have now added the SRMR and RMSEA to Table 7 (page 31).**

*(8) Page 27. Lines 477–478. “Study 3 replicated the findings of Study 2 by showing that the four-factor model of friendship preferences had a good model fit in an additional sample.”*

*Comment: On Page 23 lines 429–431 describing the results of Study 3 it states: “The model showed an acceptable fit with the exception of the relative chi-square and RMSEA (see Tables 7 and 8 for factor loadings and correlations).” Therefore, it would not be accurate to describe the model fit as “good” in the Discussion section on page 27.*

**We have changed the word "good” to “acceptable” (line 635, page 34).**

*(9) Page 29. Lines 515–517. “Finally, the FHQ dimensions, in particular intimacy, were included in an existing measure of ideal friendship standards (56) and may be relevant to social bonds formed by primate species (34).”*

*Comment: The focus of the paper is on the scale development of the FHQ questionnaire for measuring socializing preferences in humans. The reference to primate species appears to detract the focus away from the discussion of the main findings.*

**We have changed the focus to be about humans and all age groups and updated the references (line 677, page 36).**

**Reviewer 2**

- *Thanks for inviting me to review the manuscript “Friendship Habits Questionnaire: A measure of group- versus dyadic-oriented socializing preferences”. (…) As friendship is such an important part of life, a variation of assessments with distinct underlying concepts are needed to understand this complex phenome better. Therefor the authors can be complemented for their efforts and hard work to present the development of the FHQ.*

**Thank you for your kind comments. We agree that friendship is such an important part of life.**

*Despite the accolades, there are several suggestions to improve the strength of the manuscript.*

- *Abstract:
  It should be explicit that this is a new measure and that the three performed studies investigated psychometric properties and refinement of the underlying construct.*
- *It is further confusing to first read about competitiveness and later in the results about other dimensions. It might be more important to present the overall aim of all three studies.*

**We now emphasise the aim of the study in the abstract (lines 24-27, page 2). We have also tried to highlight the difference between our original questionnaire and the final product (line 34, page 2).**

- *Introduction:
  Please explain more in detail why this new measure is needed (line 61- 65 is too short)*
- *Line 68: it is definitely needed to explain how you came to the construct with the four dimensions you started with and made factor analysis.*

**We now explain in greater detail why a measure like the Friendship Habits Questionnaire is needed (lines 71 – 79, page 4). We have also added our search terminology with relevant citations (lines 80 – 82, page 4).**

- *Please insert a clear overall research question (Line 109) instead of describing the overall examination.*

**We have now added a research aim (lines 129 – 132, page 6).**

- *Line 110. Which previous findings? Please insert again references.*

**We have added references both after “previous findings” and after listing each specific trait (lines 133-136, page 6).**

- *Line 116-124: Instead of describing the organization of the three studies, I would prefer a figure (type flowchart? ) to support readers orientation.*

**We have now added a flowchart (Figure 1, lines 184-188, page 8-9). The flowchart covers information including different models and when things were removed, participant numbers and key analyses. The flowchart is color-coded to reflect what is in each study.**

- *Study 1
  Please organize each study (1-3) along research questions, hypothesis followed (as you did) by methods (participants, recruitment, material) and analysis. This would make it much easier to retrain from repetition such as in line 128 – 131.*

**We have followed your suggestion and added an aims and hypotheses section at the beginning of each study (see pages 10, 18 and 28).**

- *I propose to put the description of the FHQ with the original underlying concepts derived from other measures in the introduction (line 150 – 168). Then each of the sequential studies are shorter and easier to understand.*

**Following your suggestion, this part has been moved to the introduction (lines 141-159, page 7).**

- *Line 170 – 174: differentiate between analysis and results instead of mixing them. This is for me as reader confusing.*

**We have added an analytic strategy section to each study (see pages 12, 19-20 and 30)**

- *Table 1 is informative, but the Asterisks confusing with p>0.5. So I suggest using another graphical solution to indicate reverse-scores FHQ items.*

**Reverse-scored items have now been replaced with ∆ (see Table 1, page 15).**

- *Study 2
  See suggestion of organization of study 1.
  Line 242 -245 redundancy, instead make the aims and /or the different research questions of study 2 explicit (may be also possible to put into the flowchart/figure?)*

**We have re-written the beginning of each study adding an aims and hypotheses section (see pages 10, 18 and 28)..**

- *Line 267: Again, separate analysis and results.*

**The sections have been separated (see pages 12, 19-20 and 30).**

- *Table 4: I miss explanation of the Friendship preferences. Are these summary scores?*

**Friendship Styles (previously labelled friendship preferences) in Table 4 refers to a second-order factor assessed in a series of multi-level CFA. The description of the procedure can be found in the analytic strategy section (lines 387 to 393, page 20). Specifically, the models assessed how well our theoretical dimensions (e.g., extraversion, intimacy etc.), treated as first-order factors, relate to our underlying concept of friendship styles, treated as a second-order factor. Thus the “Friendship styles” sections of tables 4 and 7 show associations between first- and second-order factors.**

- *Table 6: the first line does not fit with the following ones. Please adjust.*

**Thank you for noticing this issue, which we have now fixed (see page 26).**

- *Study 3
  Line 412 and 416: give a methodological reason for the attention check.*

**We have added our reasoning for using the attention check (lines 550-553, page 29). Due to the length of Study 3 and after feedback from participants in Study 2, we wanted to make sure fatigue was not influencing our results.**

- *Line 412: please explain why you changed the options to describe the preference of friendship habits as compared to study 2.*

**We have added an explanation (lines 566-568, page 30). In Study 2, we saw our three original options as small, medium, and large groups. However, on reflection we were not sure if there was a difference between socializing in a group with 2 friends or 3 friends.**

- *Line 425: I suggest deleting the emotion recognition task if not explained.*

**We have deleted this sentence as suggested (line 571 – 579, page 30).**

- *General Discussion

  I disagree with the main aim to validate the FHQ. To my understanding, the three sequential studies develop the FHQ by examining and adapting the construct to achieve best validity and a small part of reliability. As validation of a measurement are somehow much more than you did (e.g. concurrent validity, age and gender validity….) , I suggest to stay here modest.*

**We have made the wording less strong (line 652, page 35).**

- *Line 540: I expect here more suggestions how to use the FHQ and practical implications. Asking directly are 4 main questions, while the FHQ has 30! Line 544 What prosper utility see the authors?*

**We now explain in greater detail the unique contribution of the FHQ compared to direct questions (lines 702-720, pages 37 - 38).**

- *Line 550: I think you should reflect on more limitations of your work. One is the participants of these studies: first their age rang is quite small and secondly the gender inequality may have an influence on your results. Please also reflect on the influence the Covid lockdown might have on the results. Another limitation is that reliability is not measured with test-retest reliability or stability over time.*

**We now acknowledge these limitations (see lines 733-740, page 38, and 761-767, pages 39-40).**

- *Again, the authors should be commended on their time and effort develop a new measurement I the field of friendship evaluation. Continued revisions will strengthen the summaries of this early stage manuscript document.*

**Thank you once again for your positive comments. We really appreciate your feedback and we hope that our corrections have addressed the concerns listed.**

**Reviewer 3**

- *First, it would be great if the authors are clearer and more consistent with what the FHQ score actually means, throughout the manuscript. For example, the title of the manuscript says "a measure of group-oriented versus dyadic-oriented socializing preferences." In Genenral Discussion, however, it says "a novel scale measuring whether a person socializes in groups."*

**High scores on the FHQ indicate that a person scores highly on traits associated with group-oriented friendship styles, thus being more likely to socialize in friendship groups. Conversely, having a low score would suggest that a person is more likely to socialise in dyads. We have tried to make this consistent throughout the paper and added further explanations at the beginning of the General Discussion (lines 652 -657, page 35). We have also switched the order throughout the paper to say group versus dyad-oriented.**

- *Relatedly, I found it unclear whether the FHQ score indicates the extent to which one prefers socializing in groups (of three people or more) or the group size that a person prefers in socialization.*

**The questionnaire is meant to estimate how likely a person is to socialize in a friendship group (versus a dyad) based on their individual traits. The items themselves refer to social behaviours or associated feelings (e.g., *I tend to be quiet when I am in larger groups of friends*, *My friends and I tell each other private things*, *I feel strong ties to a friendship group*) rather than preferences. After your comment, we realise how we have used the terms “socialize more in” and “have a preference for” as synonyms when they do not have quite the same meaning. This is, indeed, confusing and we have changed the wording throughout the text, replacing “preferences” by “friendship styles” and “socializing” behaviours. We have also changed the title to reflect this.**

- *Second, the authors mentioned that Study 1 provided the participants with the definition of friendship habits. Importantly, the definition of FH pretty much reflects the four theory-driven factors including the factor "competitiveness". It would be helpful if the authors could explain why they provided such a definition to the participants, and how it might have affected the participants' responses.*

**Our approach in Study 1 was inspired by Hinkin and Tracey (1999) who argue that a theory-driven approach to initial questionnaire development can be just as effective as a data reduction approach. We have added this to the Introduction (lines 163-172, pages 7-8), the analytic strategy of Study 1 (lines 253 – 54, page 12) and to the General Discussion as a possible limitation (lines 745-758, page 39).**

- *Third, the authors mentioned that the participants in Study 3 were provided with the descriptions of friendship interactions and example activities. I recommend the authors to explain what the descriptions were, even if it is quite brief, as it is important how the term 'friendship' was defined in this research.*

**We have provided the exact wording that was given to participants in the procedure and materials section (see lines 561-563, page 30).**
